# Circulating inflammatory markers may mediate the relationship between low carbohydrate diet and circadian rhythm in overweight and obese women

**DOI:** 10.1186/s12905-021-01240-5

**Published:** 2021-03-01

**Authors:** Atefeh Tavakoli, Atieh Mirzababaei, Forough Sajadi, Khadijeh Mirzaei

**Affiliations:** Department of Community Nutrition, School of Nutritional Sciences and Dietetics, Tehran University of Medical Sciences (TUMS), P.O. Box 14155-6117, Tehran, Iran

**Keywords:** Low carbohydrate diet, Circadian rhythm, Inflammation, Obesity

## Abstract

**Background:**

Low carbohydrate diet (LCD) can improve inflammation and obesity and also circadian rhythm disorders can lead to increased inflammation in obese individuals. The purpose of this study is to evaluate the association between adherence of LCD and circadian rhythm mediated by inflammatory markers including transforming growth factor-β (TGF-β), interleukin-1β (IL-1β) and Galectin-3 in overweight and obese women.

**Methods:**

304 women affected by overweight and obesity were enrolled. We evaluated LCD scores by Semi-quantitative food frequency questionnaire (FFQ) of 147 items. The morning-evening questionnaire (MEQ) was applied to evaluate the circadian rhythm. Biochemical parameters such as inflammatory markers and anthropometric components were assessed.

**Results:**

There was a negative significant correlation between adherence of LCD and circadian rhythm status. In other words, as the LCD scores increased, the odds of circadian rhythm disturbance in intermediate group and morning type persons decreased compared to evening type. It was showed that, IL-1β and Galectin-3 in intermediate and morning type groups, destroyed the significance of this relationship and may be considered as mediating markers.

**Conclusion:**

Adherence of LCD can improve the circadian rhythm by reducing levels of inflammatory markers and may be considered as a treatment for obesity.

## Background

Obesity is a global health threat in all over the world and is defined as the accumulation of excess fat in white adipose tissue (WAT) [1]. Obesity has been widespread in recent years throughout the world, especially in Asian countries [2]. This issue is associated with chronic and non-communicable diseases, such as cardiovascular disease, diabetes, cancers (especially breast and endometrial cancer), polycystic ovary syndrome (PCOS) in women [3, 4].

Inappropriate lifestyle, including unhealthy dietary patterns and circadian rhythm disturbances, are the possible causes for increasing the obesity trend [5]. Meal times are considered as a novel risk factor for increasing the accumulation of fat over the body [6]. Some cohort studies have demonstrated that, persons who consume a larger portion of their calories, specially carbohydrates and proteins, after midnight have higher odds of increasing levels of inflammation and becoming overweight or obese [7]. Studies have shown that, low-carbohydrate meals can lead to greater reduction in inflammatory markers and body fat mass (BFM) and metabolic syndrome risk factors, such as fasting glucose levels, triglycerides and blood pressure compared to low-fat meals [8–10]. During a period of carbohydrate restriction, ketone bodies are elevated within the plasma via hepatic production [11]. Ketone bodies act as an alternative fuel source, attenuating glucose utilization, and reducing oxidative stress and inflammation [12].

As we mentioned, circadian rhythm disorders are positively associated with obesity and weight gain. Circadian rhythm as one of the biological regulator of many processes in the body such as sleep regulation, is created by the suprachiasmatic center (SCN) of the hypothalamus in the brain and plays its role by circadian locomotor out-put cycles kaput (CLOCK) and brain and muscle arnt-like protein-1 (BMAL1) in many cells such as adipose tissue cells in the body. Adipose tissue, as an endocrine active organ, secretes different adipokines. Circadian rhythm disorders can affect adipose tissue activity and increase pro-inflammatory adipokines secretion [13]. Circadian regulation is disabled during inflammation status. Because in these conditions, the presence of endotoxins disrupt the expression of clock genes which leads to circadian rhythm disorder [14]. Although, circadian rhythm regulates the inflammatory response of healthy organisms when they first encounter a pathogen but it is lost at the molecular level once systemic inflammation ensues. This information indicates a link between circadian rhythms and inflammation [15].

TGF-β as an inflammatory markers with more than 30 related proteins have been identified as members of the TGF-β superfamily in mammals which are mostly present in liver tissue (Liver stellar cells) [16]. Galectin-3, only chimera-type galectin, has been found in the cellular nucleus and cytoplasm, at the cell surface, and in the extracellular fluid of several cell types [17] maybe closely linked to the inflammatory cascade and various disease [18]. IL-1β is a component of the mixture of pro-inflammatory cytokines that is produced by a variety of cells, including monocytes, macrophages, fibroblasts, and endothelial cells and is responsible for the inflammation occurring [19].

There is no research on the association between LCD and circadian rhythm status mediated by inflammatory markers. The correlation between inflammatory markers TGF-β, IL-1β and Galectin-3 with adherence of LCD as well as circadian rhythm disorders is not well understood. The purpose of this study, therefore, is: investigating the relationship between adherence of LCD and circadian rhythm as well as evaluating the mediating role of three inflammatory markers (TGF-β, IL-1β, Galectin-3) among women with overweight and obesity.

## Method and materials

### Study population

The sample size was computed according to the following formula: n = [(Z1 − α + Z 1 − β) × √1 − r^2^]/r)^2^ + 2), which r = 0.25, β = 0.95, and α = 0.05 then, with 95% confidence and 80% power, 304 women aged 18 to 48 years were required in this cross-sectional study. The study population was collected from across the regions of Tehran, using community-based sampling according to cluster sampling. Their body mass index (BMI) range was between 25 to 49.6 kg/m^2^. Moreover, Subjects were chosen based on the following inclusion criteria: aged 18 to 48 years, individual with overweight or obesity (BMI ≥ 25) and without menopause; exclusion criteria: regular use of medications except contraceptives (according to our investigations, the use of oral contraceptives can increase the levels of some inflammatory markers such as hs-CRP, IL-6 and TNF-α among women [20, 21]), a history of hypertension, cardiovascular disease, diabetes mellitus, liver and kidney disorders, alcohol consumption, smoking, pregnancy or lactation, menopause, chronic diseases that affect a person's diet, body weight fluctuations over the past 1 year, following patterns and special diets and non-routine diets and participants that their energy intake was less than 800 kcal or more than 4200 kcal. The protocol was accepted by the Ethical Commission at Tehran University of Medical Sciences (IR.TUMS.VCR.REC.1397.804) and all participants signed a consent form.

### Anthropometric assessment

Body composition points such as; BFM, Fat free mass (FFM), body fat percentage (BFP), visceral fat area (VFA) and obesity degree percentage (ODP); evaluated using a body composition analyzer (InBody770 scanner; InBody, Seoul, Korea). Before examining body composition, according to the instructions of the manufacture, all participants were asked to remove their metal tools, including all jewelry. womenwere also asked to take off their socks before placement on the metal plate of the device to increase the accuracy of the device. In using this device, how to stand and grab the metal handles of the device was done according to the manufacturer's instructions [22]. Weight was measured with the use of a digital scale (Seca, Hamburg, Germany) in light clothing and without shoes with precision near to 0.1 kg. Height was evaluated by a seca stadiometer, with exactness close to 0.1 cm. Waist circumference (WC) and hip circumference (HC) were measured in the smallest girth and the largest girth, respectively, with accuracy nearest to 0.1 cm. BMI was calculated as weight (kg) to height (m^2^) ratio.

### Biochemical assessment

Blood samples were taken after10–12 h of overnight fasting and the serum was centrifuged, liquated and stored at a temperature of − 80 °C. All samples were analyzed by using a single assay according to manufacturer’s protocol. All measurements were taken at the Biochemistry Laboratory of School of Health, Tehran University of Medical Sciences. Insulin level was assessed by using an enzyme-linked immunosorbent assay (ELISA) kit (Human insulin ELISA kit, DRG Pharmaceuticals, GmbH, Germany) and fasting concentration of glucose was measured by using a glucose oxidase method. Total cholesterol (T-Chole), triglyceride (TG), High-density lipoprotein cholesterol (HDL-C) and Low-density lipoprotein cholesterol (LDL-C) were evaluated by using of enzymatic approaches and related kits (Pars Azemun, Iran) and auto analyzer system. TGF-β and IL-1β were measured by HUMAN TGF-BETA and IL-1β Quantikine ELIZA kit R&D System- usa, Galectin-3 was measured by Human Galactin-3*96 T, ELIZA kit Crystal Company. We did not consider white blood cell counts as inflammatory markers in the study because many studies have shown that white blood cells-related markers of inflammation are associated with diseases such as depression [23], periodontitis [24], cardiovascular diseases such as atherosclerosis [25] and coronary artery disease [26], which are considered as factors associated with obesity is not very reasonable and has little scientific value. Investigations have shown that adherence of LCD reduces some inflammatory markers levels such as hs-CRP and IL-6 [11]. Obesity is characterized by the chronic low-grade activation of the innate immune system. In this respect, macrophage-elicited metabolic inflammation and adipocyte-macrophage interaction has a primary importance in obesity so, inflammatory markers derived from these macrophages such as IL-1β, Galectin-3 that also have interaction by TGF-β are effective in the development of obesity [27] and also as far as we have seen, in recent studies on the association of circadian or LCD with inflammation, these three inflammatory markers have been studied less than other cytokines, so we decided to select these three factors to investigate the mediating effect of inflammation.

### Circadian rhythm assessment

Circadian rhythm was evaluated by using MEQ which is a self-related and common questionnaire for assessing this rhythm status. The questionnaire consisted of 19 items that they focused on sleep pattern and waking habits and scores range from 16 to 86 points. The classification of these points is as follows: absolute evening type, which is rated 16 to 30, relative evening type for score 31 to 41, intermediate for score 42 to 58, relative morning type for score 59 to 69, and finally absolute morning type for rating 70 to 86 is considered [28]. Reliability and validity of the MEQ questionnaire were assessed in Iranian population [29].

### Calculation of the low-carbohydrate-diet score

We used semi-quantitative FFQ with 147 Iranian food items, containing a list of foods with their standard serving sizes and it assesses the usual food intake over the previous years. The reliability and validity of this questionnaire have been confirmed [30]. We calculated the LCD score for each subject by using data from completed FFQ forms by participants. Participants were divided into 11 strata based on their carbohydrates, refined grains, vegetable proteins, monounsaturated fatty acids (MUFA), n3/n6 polyunsaturated fatty acids (PUFA), expressed as a percentage of energy intake as well as fibers (gr/1000 kcal), and glycemic load (GL) intake. Dietary GL was estimated as (total glycemic index * total available carbohydrate)/100. Women in the lowest stratum of refined grains, carbohydrates and GL were given a score of 10 and those in the highest stratum were given a score of 0. For n3/n6 PUFA, MUFA, fiber, and vegetable protein intake the order of the strata was reversed. Then we summed points for the seven items to create the score was named as “low carbohydrate diet score”, that its range was between 0 (highest carbohydrate intake and lowest fat and protein intake) to 70 (lowest carbohydrate intake and highest protein and fat intake). Ultimately, the highest score indicates the highest rate of adherence to the LCD [31]

### Other variables

#### Demographic information

In this study, items such as educational status and job status were examined by using demographic questionnaire and it should be noted that we considered these variables as confounders (as quantitative variables).

#### Physical activity assessment

This item is considered as a confounder and a quantitative variable was determined by the International Physical Activity Questionnaires (IPAQ), the complex questionnaires that are designed to provide information especially for research purposes. These questionnaires can be used by person with an age range of 18 to 65 years old. Weekly physical activity was assessed by a 9 items form that was applied according metabolic equivalent (MET) scores for each type of activity. According to the IPAQ scoring protocol, MET scores were demonstrated as 3 and 6 METs for moderate activities and as 6 METs for severe activities. For reporting the total physical activity, MET scores were summed and MET-minutes per week (MET-min/wk) were reported [32, 33].

#### Stress assessment

Stress was evaluated by depression anxiety stress scales (DASS) that is a forty-two-item self-report questionnaire to evaluate stress, depression, and anxiety over the past week [34]. This item is also considered as a confounding factor and a quantitative variable.

### Statistical analyzes

The normal distribution of data was checked by Kolmogorov Smirnov test. We used mean and standard deviation to describe quantitative variables. In this study, we made quartiles from the scores of LCD and then divided the quartiles into two groups: the first group, which included the first quartile, as following a low adherence of LCD, and the second group, which included the second and third and fourth quartiles as following other groups. In this study, participants weredivided into three groups based on their obtaining of MEQ scores (16 to 41: evening type, 42 to 58: intermediate type and 59 to 86: morning type). For calculating of correlations between each variables and circadian rhythm status, we used analysis of variance (ANOVA) test. Independent sample t-test was used to check the relationship between variables and LCD status. Analysis of Covariance (ANCOVA) was then used to find the difference between the means of investigated variables across circadian rhythm and LCD, adjusted for total energy intake, physical activity and education. Associations between LCD and circadian rhythm status were assessed in Multinomial logistic regression to adjust for confounder factors including; job, FFM, physical activity and stress. We considered the group of “evening type” about circadian rhythm status and “other groups” about adherence of LCD as references. We also assessed the mediatory effects of inflammatory markers (TGF-β, IL-1β, Galectin-3) in this model. To investigate the mediating role of these inflammatory markers, we included each of them as a confounding variable separately along with the other confounders in the final model which showed a significant relationship between LCD and circadian rhythm and we considered each one that eliminated significance as a mediating factor. Statistical analyzes were performed using IBM SPSS version 25.0 (SPSS, Chicago, IL, USA). The level of significance was considered as being a P value ≤ 0.05 for all analyses.

## Result

### Study population characteristics

The characteristics of 304 women with overweight and obesity were analyzed in this study. The Mean (SD) age, BMI, LCD score, MEQ status are 36.49 (8.38) years, 31.05 (4.33) kg/m^2^, 35.01 (10.22), 50.5 (19.47); respectively (Table 1).

**Table 1:** Characteristics of the study participants

Variable	Mean ± SD	Minimum	Maximum
Age (years)	36.49 ± 8.35	18	56
Body weight (kg)	80.87 ± 12.44	59.5	136.6
Height (cm)	161.41 ± 5.92	142	179
BMI (kg/m^2^)	31.05 ± 4.33	24.2	49.6
LCD score	35.01 ± 10.22	6	58
MEQ status	50.5 ± 19.47	31	70
Sleep night	6.5 ± 1.59	1	12
Sleep day	3 ± 13.46	2	4
Stress status	7.98 ± 5.04	1	21
Physical activity (MET-minutes/week)	1202.05 ± 2085.34	40	19,194
Blood parameters			
Glucose (mmol/dl)	87.56 ± 9.6	67	137
Total cholesterol (mg/dl)	185.10 ± 35.84	104	344
TG (mg/dl)	121.81 ± 69.32	37	512
HDL (mg/dl)	46.52 ± 10.88	18	87
LDL (mg/dl)	95.06 ± 24.38	34	156
TGF-β (mg/L)	78.67 ± 48.58	32.92	494.66
IL-1β (mg/L)	2.71 ± 0.92	0.92	5.29
Galectin-3 (ng/ml)	3.96 ± 7.07	0.15	32.29
Body composition			
BFM (kg)	34.04 ± 8.69	19.4	74.2
BFP (%)	41.53 ± 5.48	15	54.3
FFM (kg)	46.8 ± 5.64	35.3	65.7
FFMI	17.93 ± 1.51	14.6	22.6
FMI	13.15 ± 3.37	6.9	26.9
WC (cm)	99.01 ± 10.05	80.1	136
VFA (cm)	163.34 ± 38.45	75.3	284.1
ODP (%)	144.09 ± 20.47	71	231

SD, Standard deviation; BMI, Body mass index; LCD, Low carbohydrate diet; MEQ, Morning-evening questionnaire; TG, Triglyceride; HDL, High density lipoprotein; LDL, Low density lipoprotein; TGF-β, Transforming growth factor β; Il-1β, Interleukin-1β; BFM, Body fat mass; BFP, Body fat percentage; FFM, Fat free mass; FFMI, Fat free mass index; FMI, Fat mass index; WC, Waist circumference; VFA, Visceral fat area; ODP, Obesity degree percentage

Based on the MEQ, 11.7% of participants were in evening type group and 58.7% were in intermediate type and 29.6% were in morning type groups. As shown in Table 2, before adjustment, the relation between age (P = 0.02), stress status (P = 0.001) and circadian rhythm status was significant. After adjustment for confounders including; total energy intake, physical activity and education through ANCOVA test the significance between age and circadian rhythm status was lost (P = 0.17) and the significance for stress status remained (P = 0.002); also other variables including; weight (P = 0.04), BFM (P = 0.03), WC (P = 0.03), VFA (P = 0.01), BMI (P = 0.05), fat mass index (FMI) (P = 0.05) and ODP (P = 0.05) became significantly associated with circadian rhythm status after adjustment. All variables mentioned, were higher in evening type group, so, the increase in obesity-related indicators was higher in women with evening disorders of circadian rhythm (Table 2).

**Table 2:** Description of characteristics among types of circadian rhythm status

Variable		Relative evening type n = 57 11.7%	Intermediate n = 159 58.7%	Relative morning type n = 88 29.6%	P value^*^	P-ANCOVA^**^
Age (years)		34.07 ± 9.12^1^	35.87 ± 8.6	38.75 ± 7.93	**0.02**	0.17
Body weight (kg)		83.3 ± 15.88	80.38 ± 11.65	77.88 ± 10.61	0.11	**0.04**
Height (cm)		162.62 ± 6	16.85 ± 5.95	160.44 ± 5.77	0.26	0.83
BMI (kg/m^2^)		31.37 ± 4.89	31.12 ± 4.24	30.28 ± 3.79	0.33	**0.05**
Sleep night		8.22 ± 1.87	7.75 ± 1.62	7.54 ± 1.59	0.22	0.24
Sleep day		12.2 ± 26.72	0.69 ± 2.79	7.77 ± 19.06	0.11	0.29
Stress status		10.92 ± 5.01	7.8 ± 4.52	6.75 ± 5.34	**0.001**	**0.002**
Physical activity (MET-minutes/week)		936.06 ± 1161.94	1087.48 ± 1665.42	1080.05 ± 1183.76	0.9	0.77
Blood parameters						
Glucose (mmol/dl)		87.47 ± 8.55	87.49 ± 10.21	86.79 ± 10.75	0.89	0.66
Total cholesterol (mg/dl)		187.69 ± 47.79	177.8 ± 31.78	186.65 ± 32.61	0.16	0.16
TG (mg/dl)		109.30 ± 49.41	122.2 ± 70.46	121.79 ± 54.23	0.64	0.73
HDL (mg/dl)		45.52 ± 12.16	46.65 ± 8.72	48.15 ± 10.87	0.48	0.75
LDL (mg/dl)		97.47 ± 28.28	95.42 ± 23	102.36 ± 21.3	0.18	0.26
TGF-β (mg/L)		73.21 ± 26.51	81.81 ± 43.3	72.89 ± 31.98	0.43	0.62
IL-1β (mg/L)		2.38 ± 0.88	2.72 ± 0.96	2.51 ± 0.95	0.59	0.41
Galectin-3 (ng/ml)		7.63 ± 10.56	5.54 ± 9	2.82 ± 4.85	0.32	0.27
Body composition						
BFM (kg)		35.27 ± 11.12	34.1 ± 8.33	32.19 ± 7.56	0.18	**0.03**
BFP (%)		41.51 ± 5.52	41.94 ± 5.37	40.87 ± 5.05	0.39	0.11
FFM (kg)		48.08 ± 5.78	46.35 ± 5.52	45.91 ± 5.93	0.23	0.39
FFMI		18.11 ± 1.41	17.9 ± 1.54	17.75 ± 1.29	0.54	0.3
FMI		13.25 ± 3.77	13.28 ± 3.72	12.52 ± 3.02	0.29	**0.05**
WC (cm)		100.67 ± 12.08	98.75 ± 9.72	96.62 ± 9.27	0.12	**0.03**
VFA (cm)		166.64 ± 45.66	164.45 ± 37.36	153.88 ± 36	0.13	**0.01**
ODP (%)		145.96 ± 22.77	144.88 ± 19.8	140.82 ± 17.66	0.31	**0.05**

N: 304; 1: Mean ± SD; BMI, Body mass index; TG, Triglyceride; HDL, High density lipoprotein; LDL, Low density lipoprotein; TGF-β, Transforming growth factor β; Il-1β, Interleukin-1β; BFM, Body fat mass; BFP, Body fat percentage; FFM, Fat free mass; FFMI, Fat free mass index; FMI, Fat mass index; WC, Waist circumference; VFA, Visceral fat area; ODP, Obesity degree percentage

^*^P-values are resulted from ANOVA test

^**^P-value reported after adjusting total energy intake, physical activity and education with ANCOVA

In Table 3, 30.3% of individuals were in low adherence of LCD group and 69.7% were in other groups and it was shown that weight (P = 0.001), height (P = 0.04), BMI (P = 0.04), BFM (P = 0.01), BFP (P = 0.03), FFM (P = 0.03), FMI (P = 0.04), WC (P = 0.03), VFA (P = 0.02), ODP (P = 0.03); were all significant and age was marginally significant (P = 0.07) with LCD status. After adjustment for confounder including; total energy intake, physical activity and education through ANCOVA test; BMI (P = 0.02), FMI (P = 0.001) and ODP (P = 0.01); remained significant and BFM and BFP (P = 0.06) and physical activity (P = 0.08) became marginally significant with LCD status and all of them were higher in low adherence of LCD group, so low carbohydrate intake can lead to decreased obesity-related indicators (Table 3).

**Table 3:** Description of characteristics among groups of adherence of LCD

Variable	Low adherence of LCD n = 79 30.3%	Other groups n = 212 69.7%	P value^*^	P-ANCOVA^**^
Age (years)	35.05 ± 8.64 ^1^	37.07 ± 8.41	0.07	0.86
Body weight (kg)	84.19 ± 14.04	79.43 ± 11.25	**0.001**	0.21
Height (cm)	162.37 ± 5.61	160.88 ± 6.1	**0.04**	0.2
BMI (kg/m^2^)	32 ± 5.06	30.69 ± 3.96	**0.04**	**0.02**
Sleep night	7.91 ± 1.71	7.56 ± 1.54	0.11	0.62
Sleep day	5.79 ± 17.12	3.42 ± 12.09	0.49	0.79
Stress status	8.5 ± 4.96	7.75 ± 5.06	0.27	0.54
Physical activity (MET-minutes/week	1512.32 ± 2602.91	1096.31 ± 1901.06	0.24	0.08
Blood parameters				
Glucose (mmol/dl)	87.96 ± 9.41	87.49 ± 9.72	0.7	0.28
Total cholesterol (mg/dl)	182.07 ± 31.1	185.96 ± 38.04	0.41	0.95
TG (mg/dl)	124.56 ± 74.99	121.29 ± 68.94	0.74	0.77
HDL (mg/dl)	46.21 ± 9.48	46.93 ± 11.34	0.64	0.49
LDL (mg/dl)	93.61 ± 22.15	95.2 ± 25.27	0.65	0.73
TGF-β (mg/L)	72.71 ± 22.09	80.03 ± 54.1	0.21	0.81
IL-1β (mg/L)	2.82 ± 0.84	2.68 ± 0.96	0.59	0.41
Galectin-3 (ng/ml)	4.89 ± 8.35	3.66 ± 6.6	0.7	0.67
Body composition				
BFM (kg)	36.44 ± 10.33	33.1 ± 10.8	**0.01**	0.06
BFP (%)	42.15 ± 6.45	41.28 ± 5.15	**0.03**	0.06
FFM	47.94 ± 5.44	46.34 ± 5.58	**0.03**	0.6
FFMI	18.16 ± 1.58	17.85 ± 1.46	0.12	0.54
FMI	13.89 ± 3.91	12.88 ± 3.14	**0.04**	**0.001**
WC (cm)	101.03 ± 10.86	98.19 ± 9.62	**0.03**	0.52
VFA (cm)	171.61 ± 42.64	159.94 ± 36.52	**0.02**	0.1
ODP (%)	148.84 ± 23.6	142.36 ± 19	**0.03**	**0.01**

N: 304; 1: Mean ± SD; BMI, Body mass index; TG, Triglyceride; HDL, High density lipoprotein; LDL, Low density lipoprotein; protein; TGF-β, Transforming growth factor β; Il-1β, Interleukin-1β; BFM, Body fat mass; BFP, Body fat percentage; FFM, Fat free mass; FFMI, Fat free mass index; FMI, Fat mass index; WC, Waist circumference; VFA, Visceral fat area; ODP, Obesity degree percentage

^*^P-values are resulted from independent t sample test

^**^P-value reported after adjusting total energy intake, physical activity and education with ANCOVA

### Association of the LCD status and circadian rhythm status

A negative significant association between low adherence of LCD status compare to other groups, and circadian rhythm was observed in crude model about intermediate (OR = 0.38, 95% CI = 0.16 to 0.9, P = 0.02) and morning type (OR = 0.32, 95% = 0.12 to 0.84, P = 0.02). In the adjusted model according to job, FFM, physical activity, stress as covariates, the correlation remained significant in intermediate (OR = 0.33, 95% CI = 0.12 to 0.89, P = 0.02) and also in morning group (OR = 0.27, 95% CI = 0.08 to 0.86, P = 0.02) compare to evening type (Table 4).

**Table 4:** Relation between adherence LCD and circadian rhythm status

	LCD status			
	Low adherence^a^			Other groups*^b^
	OR (95%CI)	β ± SE	P	
Evening type*				
Intermediate				
Crude	0.38 (0.16–0.9)	− 0.95 ± 0.43	**0.02**	
Adjusted	0.33 (0.12–0.89)	− 1.1 ± 0.5	**0.02**	
Morning type				
Crude	0.32 (0.12–0.84)	1.28 ± 0.29	**0.02**	
Adjusted	0.08–0.86))0.27	− 1.28 ± 0.58	**0.02**	

OR, odds ratio; CI, confidence interval; SE, standard error; N: 304; Multinominal logistic regression; Crude model and adjusted model to job, fat free mass, physical activity, stress as covariates

^*^Consider as reference

^a^Low adherence includes first quartile

^b^Other groups include second, third and fourth quartiles

### Assess the mediating role of inflammatory markers

We associated the effect of inflammatory markers including; TGF-β, IL-β and Galectin-3; as mediatory markers for the significant relationship between LCD and circadian rhythm. By including each of these inflammatory markers as a confounding variable along with the other confounders in the final model, which showed a significant relationship between LCD and circadian rhythm, we observed that two inflammatory markers including; IL-1β (OR = 0.33, 95% CI = 0.02 to 4.49, P = 0.67) and Galectin-3 (P = 0.21, 95% CI = 0.02 to 2.06, P = 0.18) in intermediate and also in morning type ( IL-1β (OR = 0.36, 955 CI = 0.02 to 6.04, P = 0.49) and Galectin-3 (OR = 0.17, 95% CI = 0.01 to 1.95, P = 0.4)), eliminated this significance so they can be considered as mediatory markers (Table 5).

**Table 5:** The association of the mediating effect of some inflammatory markers

	LCD status		
	OR (95%CI)	β ± SE	P
Evening type*			
Intermediate			
IL-1β	0.33 (0.02–4.49)	− 1.10 ± 1.33	**0.67**
Galectin-3	0.21 (0.02–2.06)	− 1.52 ± 1.14	**0.18**
TGF-β	0.12 (0.03–0.47)	− 2.12 ± 0.69	0.004
Morning type			
IL-1β	0.36 (0.02–6.04)	− 1 ± 1.46	**0.49**
Galectin-3	0.17 (0.01–1.95)	− 1.76 ± 1.24	**0.4**
TGF-β	0.11 (0.02–0.53)	− 2.13 ± 0.77	0.002

OR, Odds ratio; CI, confidence interval; SE, standard error; Il-1β, Interleukin-1β; TGF-β, Transforming growth factor β; N: 304; Multinominal logistic regression adjusted model to job, fat free mass, physical activity, stress as covariates in addition to the inflammatory markers

^*^Consider as reference

## Discussion

This cross-sectional study was the first study, investigated the association between the adherence of LCD and circadian rhythm status and also examines the mediating role of three inflammatory markers (TGF-β, IL-1β, Galectin-3) among overweight and obese women.

In this study, there was a negative significant correlation between circadian rhythm status and adherence of LCD. In other words, as the LCD scores increased (Indicates increased adherence to a LCD), the odds of circadian rhythm disturbance in intermediate group and morning type person compared to the evening type person, decreased.. Moreover in this study, we investigated that two inflammatory markers including; IL-1β and Galectin-3 play mediatory roles in the association between LCD and circadian rhythm status.

Rohit Sane et al., demonstrated that combination of LCD and obesity management procedures caused significant reduction in body weight, BMI and WC, without any adverse effect in obese people [35]. One study observed that morning carbohydrate consumption was associated with improvement of lipid and glycemic profiles. This finding supports that a higher proportion of carbohydrates is made in the first half of the day can lead to better nutritional and health status. There is evidence that carbohydrate oxidation is decreased at later circadian phases so consuming carbohydrates after midnight can lead to fat accumulation. It is not yet possible to explain exactly why the intake of macronutrients and the body's biological clock are related [36–38].

LCD reduces glucose supply to the liver, muscles and brain, leading to limited glucose availability and storage as glycogen. By restricting access to glucose, glucose-producing endogenous process called gluconeogenesis will be activated in the body. This process can not cover the body's need for glucose as the primary fuel for cells, so ketone bodies will be produced as an alternative source of glucose. In this condition, insulin secretion and consequently fat and glucose storages will be reduced. Therefore, the rate of weight gain and inflammation will decrease due to the reduction in the amount of fat cells storage [39, 40]. High glucose levels after high carbohydrate meals, promote the activation of innate immune cells and the release of pro-inflammatory cytokines. Adherence of LCD because of the reducing in fasting glucose levels can improve the inflammation status and treat obesity as a chronic inflammation [10, 41–43]. Adherence of LCD reduces some inflammatory markers levels such as hs-CRP, and increases the concentration of adiponectin as an anti-inflammatory marker (36–37). Dietary interventions such as LCD has shown some improvements in endogenous antioxidant capacity which may be used as a method for decreasing markers of oxidative stress and inflammation [44]. A study has demonstrated that reduction in dietary carbohydrate intake significantly improved some pro-inflammatory markers such as IL-6, IL-8, TNF-α [45].

Delays in food intake especially at dinner are related to obesity, metabolic disorders and increased values of inflammatory markers in obese persons [7]. The results of a recent study among obese persons show that the timing of dinner is a relevant factor in obesity. Late dinner eaters (that was, participants with circadian rhythm disorders at night) were 2.1-times more likely to be overweight/obese than early eaters, and had significantly higher odds of increasing levels of inflammatory markers including IL6 and hs-CRP [46, 47]. One of the negative consequences of circadian rhythm disorder may be the dysregulation of the immune system. Changes of the sleep/wake cycle affect the number of circulating lymphocytes and Natural killer (NK) cells in humans [48], as well as increased inflammatory markers such as IL-6, hs-CRP, and Tumor necrosis factor-a (TNF-a) [49]. The results of some studies are as follows that shift-workers that often have circadian rhythm disorders, especially at night, consume more calories, fat, protein, carbohydrates, and sweets with lower vegetable and fruit consumption and this may be the reason for the increased levels of inflammatory markers in these persons [50]. Based on the available evidence, it can be stated that stimulation of the innate immune system can alter the expression of several circadian clock genes such as Per1 and Per2 through pro-inflammatory markers [51]. Circadian rhythm can also regulate the immune system and its regulation effects are for many immune markers, including IL-2, IL-10, IL-6, IL-1β, TNF-α [51, 52]. More over studies indicated that appetite-regulating hormones such as reducing leptin secretion and increasing ghrelin levels would be influenced by circadian rhythm disorders that can stimulate the appetite and food intake and induce obesity incidence. Furthermore decreased melatonin levels can also lead to increased pro-inflammatory cytokines secretion in subjects with circadian rhythm disorders [53–55].

This study was the first study to investigate the relationship between LCD and circadian rhythm mediated by inflammatory markers and it may represent as a possible novel insight to monitor and treat obesity and its complications. We evaluated the association between many body composition indicators and circadian rhythm, as well as LCD over the study. In addition to measuring the relationship between LCD and circadian rhythm status, we also examined the mediating role of inflammatory markers which have not generally been investigated in previous studies. Despite increasing attention to women's health in recent years, studies on this group of society are still insufficient, so we selected the women as a target group to provide information for improving their health. We tried to select and adjust the confounding factors socio-economically because circadian rhythm has a strong correlation with such factors [56], so it makes the results more acceptable. However there were a number of limitations; since the study was a cross-sectional study, causality cannot be proven, only correlation. The authors’ suggestions for further studies include to work with larger sample or to carry out experimental studies to check this association.

## Conclusion

In this study, a significant relationship between adherence of LCD and circadian rhythm status was found, and it was shown that adherence of this dietary pattern reduced disorders about circadian rhythm of intermediate and morning type groups. Also it was shown that two inflammatory markers such as IL-1β and Galectin-3 had mediatory role in this relationship. So, having a LCD may improve the circadian rhythm by reducing the level of inflammatory markers and it can be considered as a treatment for overweight and obesity status.

## Data Availability

The data that support the findings of this study are available from Dr.Khadijeh Mirzaei but restrictions apply to the availability of these data, which were used under license for the current study, and so are not publicly available. Data are however available from the authors upon reasonable request and with permission of Dr.Khadijeh Mirzaei.

## Abbreviations

LCD: Low carbohydrate diet
TGF-β: Transforming growth factor-β
IL-1β: Interleukin-1β
FFQ: Food frequency questionnaire
MEQ: Morning-evening questionnaire
WAT: White adipose tissue
PCO: Polycystic ovary syndrome
SCN: Supra-chiasmatic center
CLOCK: Circadian loco-motor out-put cycles kaput
BMAL 1: Muscle arnt-like protein-1
BMI: Body mass index
BFM: Body fat mass
FFM: Fat free mass
BFP: Body fat percentage
VFA: Visceral fat area
ODP: Obesity degree percentage
WC: Waist circumference
HC: Hip circumference
FMI: Fat mass index
ELISA: Enzyme-linked immunosorbent assay
T-chole: Total cholesterol
TG: Triglyceride
HDL: High-density lipoprotein cholesterol
LDL: Low-density lipoprotein cholesterol
PUFA: Polyunsaturated fatty acids
GL: Glycemic load
IPAQ: International Physical Activity Questionnaires
MET: Metabolic equivalent
DASS: Depression anxiety stress scales
ANOVA: Analysis of variance
ANCOVA: Analysis of Covariance
TNF-a: Tumor necrosis factor-a
NK: Natural killer

## References

[1] Lespessailles E, Toumi H (2017). Vitamin D alteration associated with obesity and bariatric surgery. Exp Biol Med.

[2] Saeidlou SN, Vahabzadeh D, Babaei F, Vahabzadeh Z (2017). Seasonal variations of vitamin D and its relation to lipid profile in Iranian children and adults. J Health Popul Nutr.

[3] Hedley AA, Ogden CL, Johnson CL, Carroll MD, Curtin LR, Flegal KM (2004). Prevalence of overweight and obesity among US children, adolescents, and adults, 1999–2002. JAMA.

[4] Hu FB (2003). Overweight and obesity in women: health risks and consequences. J Women's Health.

[5] Hales CM, Carroll MD, Fryar CD, Ogden CL. Prevalence of obesity among adults and youth: United States, 2015–2016. 2017. 29155689

[6] Mattson MP, Allison DB, Fontana L, Harvie M, Longo VD, Malaisse WJ, Mosley M, Notterpek L, Ravussin E, Scheer FA (2014). Meal frequency and timing in health and disease. Proc Natl Acad Sci.

[7] Xiao Q, Garaulet M, Scheer FA (2019). Meal timing and obesity: Interactions with macronutrient intake and chronotype. Int J Obes.

[8] Gjuladin-Hellon T, Davies IG, Penson P, Amiri Baghbadorani R (2019). Effects of carbohydrate-restricted diets on low-density lipoprotein cholesterol levels in overweight and obese adults: a systematic review and meta-analysis. Nutr Rev.

[9] Lu M, Wan Y, Yang B, Huggins CE, Li D (2018). Effects of low-fat compared with high-fat diet on cardiometabolic indicators in people with overweight and obesity without overt metabolic disturbance: a systematic review and meta-analysis of randomised controlled trials. Br J Nutr.

[10] Noakes M, Foster PR, Keogh JB, James AP, Mamo JC, Clifton PM (2006). Comparison of isocaloric very low carbohydrate/high saturated fat and high carbohydrate/low saturated fat diets on body composition and cardiovascular risk. Nutr Metab.

[11] Waldman HS, Shepherd BD, Egan B, McAllister MJ (2020). Exogenous ketone salts do not improve cognitive performance during a dual-stress challenge. Int J Sport Nutr Exerc Metab.

[12] Miller VJ, LaFountain RA, Barnhart E, Sapper TS, Short J, Arnold WD, Hyde PN, Crabtree CD, Kackley ML, Kraemer WJ (2020). A Ketogenic diet combined with exercise alters mitochondrial function in human skeletal muscle while improving metabolic health. Am J Physiol Endocrinol Metab.

[13] Allebrandt KV, Roenneberg T (2008). The search for circadian clock components in humans: new perspectives for association studies. Braz J Med Biol Res.

[14] Haimovich B, Calvano J, Haimovich AD, Calvano SE, Coyle SM, Lowry SF (2010). In vivo endotoxin synchronizes and suppresses clock gene expression in human peripheral blood leukocytes. Crit Care Med.

[15] Bellet MM, Deriu E, Liu JZ, Grimaldi B, Blaschitz C, Zeller M, Edwards RA, Sahar S, Dandekar S, Baldi P (2013). Circadian clock regulates the host response to Salmonella. Proc Natl Acad Sci.

[16] Bissell DM, Roulot D, George J (2001). Transforming growth factor β and the liver. Hepatology.

[17] Jeon S-B, Yoon HJ, Chang CY, Koh HS, Jeon S-H, Park EJ (2010). Galectin-3 exerts cytokine-like regulatory actions through the JAK–STAT pathway. J Immunol.

[18] Venkatraman A, Hardas S, Patel N, Singh Bajaj N, Arora G, Arora P (2018). Galectin-3: an emerging biomarker in stroke and cerebrovascular diseases. Eur J Neurol.

[19] Coccia M, Harrison OJ, Schiering C, Asquith MJ, Becher B, Powrie F, Maloy KJ (2012). IL-1β mediates chronic intestinal inflammation by promoting the accumulation of IL-17A secreting innate lymphoid cells and CD4+ Th17 cells. J Exp Med.

[20] van Rooijen M, Hansson L, Frostegård J, Silveira A, Hamsten A, Bremme K (2006). Treatment with combined oral contraceptives induces a rise in serum C-reactive protein in the absence of a general inflammatory response. J Thromb Haemost.

[21] Divani AA, Luo X, Datta YH, Flaherty JD, Panoskaltsis-Mortari A: Effect of oral and vaginal hormonal contraceptives on inflammatory blood biomarkers. *Mediators of inflammation* 2015, 2015. 10.1155/2015/379501 PMC4378601 25861161

[22] Mirzababaei A, Sajjadi SF, Ghodoosi N, Pooyan S, Arghavani H, Yekaninejad MS, Mirzaei K (2019). Relations of major dietary patterns and metabolically unhealthy overweight/obesity phenotypes among Iranian women. Diab Metab Syndrome Clin Res Rev.

[23] Beydoun M, Beydoun H, Dore G, Canas J, Fanelli-Kuczmarski M, Evans M, Zonderman A (2016). White blood cell inflammatory markers are associated with depressive symptoms in a longitudinal study of urban adults. Transl Psychiatry.

[24] Loos BG (2005). Systemic markers of inflammation in periodontitis. J Periodontol.

[25] Chrysohoou C, Pitsavos C, Panagiotakos DB, Skoumas J, Stefanadis C (2004). Association between prehypertension status and inflammatory markers related to atherosclerotic disease: the ATTICA Study. Am J Hypertens.

[26] Kounis NG, Soufras GD, Tsigkas G, Hahalis G (2015). White blood cell counts, leukocyte ratios, and eosinophils as inflammatory markers in patients with coronary artery disease. Clin Appl Thromb Hemost.

[27] Engin AB. Adipocyte-macrophage cross-talk in obesity. In: Obesity and lipotoxicity. Springer; 2017, pp. 327–343. 10.1007/978-3-319-48382-5_14 28585206

[28] Shahid A, Wilkinson K, Marcu S, Shapiro CM. STOP, THAT and one hundred other sleep scales. Springer; 2012.

[29] Rahafar A, Randler C, Díaz-Morales JF, Kasaeian A, Heidari Z (2017). Cross-cultural validity of morningness–eveningness stability scale improved (MESSi) in Iran, Spain and Germany. Chronobiol Int.

[30] Mirmiran P, Esfahani FH, Mehrabi Y, Hedayati M, Azizi F (2010). Reliability and relative validity of an FFQ for nutrients in the Tehran lipid and glucose study. Public Health Nutr.

[31] Eslamian G, Mirmiran P, Asghari G, Hosseini-Esfahani F, Youzbashian E, Azizi F. Low carbohydrate diet score does not predict metabolic syndrome in children and adolescents: Tehran Lipid and Glucose Study. Arch Iran Med. 2014; 17(6):0–0. 24916527

[32] Craig CL, Marshall AL, Sjöström M, Bauman AE, Booth ML, Ainsworth BE, Pratt M, Ekelund U, Yngve A, Sallis JF (2003). International physical activity questionnaire: 12-country reliability and validity. Med Sci Sports Exerc.

[33] Moghaddam MB, Aghdam FB, Jafarabadi MA, Allahverdipour H, Nikookheslat SD, Safarpour S (2012). The Iranian Version of International Physical Activity Questionnaire (IPAQ) in Iran: content and construct validity, factor structure, internal consistency and stability. World Appl Sci J.

[34] Brown TA, Chorpita BF, Korotitsch W, Barlow DH (1997). Psychometric properties of the Depression Anxiety Stress Scales (DASS) in clinical samples. Behav Res Ther.

[35] Sane R, Amin D, Koli V, Kore R, Paranjpe S, Mandole R (2019). Impact of low-carbohydrate diet (LCD) and obesity management program on obese patients. Eur J Prev Med.

[36] McHill AW, Phillips AJ, Czeisler CA, Keating L, Yee K, Barger LK, Garaulet M, Scheer FA, Klerman EB (2017). Later circadian timing of food intake is associated with increased body fat. Am J Clin Nutr.

[37] Morris CJ, Yang JN, Garcia JI, Myers S, Bozzi I, Wang W, Buxton OM, Shea SA, Scheer FA (2015). Endogenous circadian system and circadian misalignment impact glucose tolerance via separate mechanisms in humans. Proc Natl Acad Sci.

[38] Bandin C, Scheer F, Luque A, Avila-Gandia V, Zamora S, Madrid J, Gomez-Abellan P, Garaulet M (2015). Meal timing affects glucose tolerance, substrate oxidation and circadian-related variables: a randomized, crossover trial. Int J Obes.

[39] Van Wyk H, Davis R, Davies J (2016). A critical review of low-carbohydrate diets in people with type 2 diabetes. Diabet Med.

[40] Hall K (2017). A review of the carbohydrate–insulin model of obesity. Eur J Clin Nutr.

[41] Dasu MR, Devaraj S, Zhao L, Hwang DH, Jialal I (2008). High glucose induces toll-like receptor expression in human monocytes: mechanism of activation. Diabetes.

[42] Giannella A, Radu CM, Franco L, Campello E, Simioni P, Avogaro A, de Kreutzenberg SV, Ceolotto G (2017). Circulating levels and characterization of microparticles in patients with different degrees of glucose tolerance. Cardiovasc Diabetol.

[43] Norseen J, Hosooka T, Hammarstedt A, Yore MM, Kant S, Aryal P, Kiernan UA, Phillips DA, Maruyama H, Kraus BJ (2012). Retinol-binding protein 4 inhibits insulin signaling in adipocytes by inducing proinflammatory cytokines in macrophages through a c-Jun N-terminal kinase-and toll-like receptor 4-dependent and retinol-independent mechanism. Mol Cell Biol.

[44] Milder J, Patel M (2012). Modulation of oxidative stress and mitochondrial function by the ketogenic diet. Epilepsy Res.

[45] Waldman HS, Heatherly AJ, Killen LG, Hollingsworth A, Koh Y, O’Neal EK. A 3-week, low-carbohydrate, high-fat diet improves multiple serum inflammatory markers in endurance-trained males. J Strength Cond Res; 2020. 10.1519/JSC.0000000000003761 32826835

[46] Martínez-Lozano N, Tvarijonaviciute A, Ríos R, Barón I, Scheer FA, Garaulet M (2020). Late eating is associated with obesity, inflammatory markers and circadian-related disturbances in school-aged children. Nutrients.

[47] Marti A, Morell-Azanza L, Rendo-Urteaga T, García-Calzón S, Ojeda-Rodríguez A, Martín-Calvo N, Moreno-Aliaga M, Martínez J, Azcona-San Julián M (2018). Serum and gene expression levels of CT-1, IL-6, and TNF-α after a lifestyle intervention in obese children. Pediatr Diabetes.

[48] Hui L, Hua F, Diandong H, Hong Y (2007). Effects of sleep and sleep deprivation on immunoglobulins and complement in humans. Brain Behav Immun.

[49] Mullington JM, Haack M, Toth M, Serrador JM, Meier-Ewert HK (2009). Cardiovascular, inflammatory, and metabolic consequences of sleep deprivation. Prog Cardiovasc Dis.

[50] Lowden A, Moreno C, Holmbäck U, Lennernäs M, Tucker P: Eating and shift work—effects on habits, metabolism, and performance. Scand J Work Environ Health 2010; 150–162. 10.5271/sjweh.2898 20143038

[51] Coogan AN, Wyse CA (2008). Neuroimmunology of the circadian clock. Brain Res.

[52] Hayashi M, Shimba S, Tezuka M (2007). Characterization of the molecular clock in mouse peritoneal macrophages. Biol Pharm Bull.

[53] Cizza G, Requena M, Galli G, de Jonge L (2011). Chronic sleep deprivation and seasonality: implications for the obesity epidemic. J Endocrinol Invest.

[54] McEwen BS (2006). Sleep deprivation as a neurobiologic and physiologic stressor: allostasis and allostatic load. Metabolism.

[55] Nieminen P, Mustonen A-M, Asikainen J, Hyvärinen H (2002). Seasonal weight regulation of the raccoon dog (Nyctereutes procyonoides): interactions between melatonin, leptin, ghrelin, and growth hormone. J Biol Rhythms.

[56] Kinjo K, Sato H, Sato H, Shiotani I, Kurotobi T, Ohnishi Y, Hishida E, Nakatani D, Ito H, Koretsune Y (2001). Circadian variation of the onset of acute myocardial infarction in the Osaka area, 1998–1999. Jpn Circ J.

